# Predictor species: Improving assessments of rare species occurrence by modeling environmental co‐responses

**DOI:** 10.1002/ece3.6096

**Published:** 2020-03-02

**Authors:** Peter R. Thompson, William F. Fagan, Phillip P. A. Staniczenko

**Affiliations:** ^1^ Department of Biology University of Maryland College Park MD USA; ^2^ Department of Biological Sciences University of Alberta Edmonton AB Canada; ^3^ National Socio‐Environmental Synthesis Center (SESYNC) Annapolis MD USA; ^4^ Present address: Department of Biology Brooklyn College City University of New York New York NY USA

**Keywords:** Bayesian networks, community ecology, peat bogs, species distribution models

## Abstract

Designing an effective conservation strategy requires understanding where rare species are located. Because rare species can be difficult to find, ecologists often identify other species called conservation surrogates that can help inform the distribution of rare species. Species distribution models typically rely on environmental data when predicting the occurrence of species, neglecting the effect of species' co‐occurrences and biotic interactions. Here, we present a new approach that uses Bayesian networks to improve predictions by modeling environmental co‐responses among species. For species from a European peat bog community, our approach consistently performs better than single‐species models and better than conventional multi‐species approaches that include the presence of nontarget species as additional independent variables in regression models. Our approach performs particularly well with rare species and when calibration data are limited. Furthermore, we identify a group of “predictor species” that are relatively common, insensitive to the presence of other species, and can be used to improve occurrence predictions of rare species. Predictor species are distinct from other categories of conservation surrogates such as umbrella or indicator species, which motivates focused data collection of predictor species to enhance conservation practices.

## Graphical abstract

It is difficult to protect rare species from extinction because of their sparse distributions and specialized habitat preferences. Here, we use Bayesian networks to accurately predict the occurrence of rare species, providing a solution to an ever‐important conservation problem.

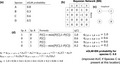

## 1 INTRODUCTION

Species distribution models (SDMs) are widely used in ecology to predict the geographical ranges of individual species (Allouche, Steinitz, Rotem, Rosenfeld, & Kadmon, 2008; Booth, Nix, Busby, & Hutchinson, 2014; Elith et al., 2006; Guisan & Thuiller, 2005; Pearson et al., 2014; Peterson, Soberón, & Pearson, 2011), and multiple SDMs can be interpreted together to estimate the composition of an ecological community at a particular location (Cassini, 2011; Kissling et al., 2012; Thuiller, Pollock, Gueguen, & Münkemüller, 2015). SDMs are also used to aid in the conservation of rare species that occur at relatively few locations compared to other species in the community (Chen & Peterson, 2002; Marcer, Sáez, Molowny‐Horas, Pons, & Pino, 2013; Rivera, López‐Quílez, & Blangiardo, 2018). Because rare species often have specialized habitat preferences (Spitale, 2012) and are harder to detect (Hannon, Cotterill, & Schmiegelow, 2003), protecting areas where rare species are known to occur or, more realistically, are expected to occur is critical for preserving the Earth's biodiversity (Lawler, White, Sifneos, & Master, 2003). However, protecting the wrong areas due to model inaccuracy is a costly mistake that does little to promote the survival of rare and threatened species (Akçakaya et al., 2017).

The growing desire and potential for SDMs to make predictions at smaller spatial scales has led to an integration of ideas from macroecology and community ecology (Eaton et al., 2018; Staniczenko, Sivasubramaniam, Suttle, & Pearson, 2017). Ecologists initially made predictions using environment‐only SDMs that included only abiotic variables like temperature and rainfall (Pearson & Dawson, 2003), but soon recognized that incorporating dependencies among species was necessary to explain empirical distribution patterns (Araújo & Luoto, 2007; Fordham et al., 2013; Ockendon et al., 2014; Schmitz, Post, Burns, & Johnston, 2003; Soberón, 2007; Ward et al., 2015). Recent work has explored a variety of approaches to modeling such dependencies in SDMs (Fernandes et al., 2013; Giannini, Chapman, Saralva, Santos, & Blesmeijer, 2013; Hollings, Robinson, Andel, Jewell, & Burgman, 2017; Lany, Zarnetske, Gouhier, & Menge, 2017; Meier et al., 2010; Pellissier et al., 2010, 2013; le Roux, Pellissier, Wisz, & Luoto, 2014; Trainor & Schmitz, 2014; Trainor, Schmitz, Ivan, & Shenk, 2014), and a simple yet successful strategy involves modeling the occurrence of a target species using the presence or absence of additional, nontarget species as independent variables in generalized linear models (GLMs) (Giannini et al., 2013; Godsoe & Harmon, 2012) and maximum entropy models (Araújo, Marcondes‐Machado, & Costa, 2014). However, this strategy has not always improved results; for example, predictions for rare species from a plant community in Britain were less accurate with multi‐species models than with single‐species versions of two machine‐learning methods (Chapman & Purse, 2011). A recent study compared different random forest models (a machine‐learning‐based method) and concluded that single‐species models yielded more accurate predictions than multi‐species models for binary response data (Henderson, Ohmann, Gregory, Roberts, & Zald, 2014). A more comprehensive approach to modeling shared environmental co‐responses involves joint species distribution models (Ovaskainen, Hottola, & Siitonen, 2010; Pollock et al., 2014), but calibrating these models requires extensive species co‐occurrence data that can be time‐consuming and labor‐intensive to collect.

Bayesian networks (BNs) offer a balanced approach to modeling how the presence of a species is affected by the presence or absence of other species (Eaton et al., 2018). While other species distribution models rely on categorizing or weighing each different type of interspecific relationship (Anderson, 2017), BNs offer a mathematical framework that can be much simpler: Interspecific relationships are represented as conditional dependencies between species, with the presence of one species potentially increasing or decreasing the occurrence probability of another species. As with other multi‐species SDMs, our approach attempts to improve predictions of an individual species' geographical distribution by accounting for the species' fundamental niche (the area where it could hypothetically occur given only environmental conditions (Soberón & Arroyo‐Peña, 2017)) and its realized niche (the actual area where it can be found, given interspecific interactions (Soberón & Arroyo‐Peña, 2017)). With an SDM involving BNs, the BN component is used to adjust “prior” probabilities of species occurrence generated by environment‐only models to produce “posterior” probabilities that also reflect the effect of biotic interactions and other interspecific relationships among species.

Here, our goal is to improve assessments of species occurrence at specific locations, especially for rare species, by including information on species' environmental co‐responses in SDM‐like predictive models. We compare the performance of three types of model: (a) environment‐only GLMs (“eGLM”); (b) multi‐species GLMs that include the presence or absence of nonfocal (i.e., nontarget) species as additional independent variables (“sGLM”); and (c) a new approach that combines probabilities from the eGLM with a BN that represents strong environmental co‐responses among species (“eGLM + BN”). We compare these three models to an approach based on joint species distribution modeling that provides an upper bound to model accuracy because it requires much more input data for calibration.

We test models using data from a European peat bog community (Robroek et al., 2017). Based on a BN for the peat bog community, we identify a group of “predictor species” that are useful for improving predictions of rare species occurrence. We suggest that predictor species could function as conservation surrogates, that is, species that are used to facilitate the management or protection of another species (Caro & O'Doherty, 1999). To this end, predictor species complement existing categories of conservation surrogates (Andelman & Fagan, 2000) such as umbrella species (typically found at many locations (Fleishman, Blair, & Murphy, 2001)) and indicator species (typically found at locations with high species richness (Azeria et al., 2009)).

## 2 MATERIAL AND METHODS

### 2.1 Data

We tested our approach using data on a peat bog community of 54 plant species at 56 locations across Europe (Robroek et al., 2017). Data were collected during the summer months of multiple years, and all but the least common species (those occurring at less than five of the 56 locations) were included in the data set (Robroek et al., 2017). Some groups (such as lichens) were not identified to the species level because of time constraints and identification difficulties (Robroek et al., 2017). Of nine available environmental variables, we included four in generalized linear models: mean annual temperature; mean annual precipitation; latitude; and temperature seasonality (measured as the difference between the warmest and coolest month in a given year). These four variables had the highest average correlations with species occurrence and were not highly correlated with each other (see Appendix S1 for more details on our choice of variables). Because our goal was to develop models for predicting the occurrence of individual species at specific locations, we converted species abundance at each location to a binary measure of presence or absence (i.e., any species with abundance over 0 was considered to be present), which we used as a dependent variable for calibrating and testing models.

Despite the relatively small number of locations, the peat bog data set has three properties that make it especially valuable for our analysis. First, the peat bog data set includes confirmed presences and absences for each species at each location, unlike many larger data sets that usually only include confirmed presences. As we are using logistic regression models, it is preferable to use confirmed absences to calibrate models rather than the assumption that the lack of an observed presence can be considered an absence. Second, the species from the peat bog community are not only closely related genetically (implying that environmental co‐responses are likely) but also live and interact in a physically close manner (implying that they may develop biotic interactions that affect their larger scale distributions)—taken together, these two features provide strong motivation for modeling the effect of interspecific relationships on geographical distributions. Third, the 56 locations are geographically dispersed enough to provide significant differences in environmental conditions among locations; so even though the absolute number of locations is relatively low, there is still sufficient variance to allow models to discriminate between the environmental preferences of species (Wisz et al., 2008).

### 2.2 Modeling occurrence predictions using only environmental variables (eGLM)

We used generalized linear models (Das & Dey, 2006; Vasconcelos, Le Pape, Costa, & Cabral, 2013) to make environment‐only predictions for the species in the peat bog community. The eGLM only included environmental data in its set of independent variables, with the presence or absence of a focal species at a specific location as the dependent variable:

(1) $$Y_{\mathrm{ij}}∼T_{j}+P_{j}+V_{j}^{T}+L_{j}$$

where $Y_{\mathrm{ij}}$ is the presence or absence of species *i* at location *j*; and $T_{j}$ is mean annual temperature, $P_{j}$ is mean annual precipitation, $V_{j}^{T}$ is temperature seasonality, and $L_{j}$ is latitude, at location *j* (see Table S1 and Table S2 for more on the choice of these variables). We used a logit link function between independent and dependent variables. Adding quadratic and interaction terms to the eGLM did not improve model performance (see Appendix S1).

### 2.3 Estimating environmental co‐responses among species

To develop models that incorporated the occurrence of nonfocal species, we constructed a correlation matrix describing the strength of all possible interspecific relationships in the peat bog community. First, we computed the Pearson correlation between the presence or absence of each pair of species across the 56 locations. The result was a symmetric 54‐by‐54 species correlation matrix with ones on the leading diagonal. We then set these ones to zero and specified a threshold value to convert all off‐diagonal entries to 0, 1, or −1, depending on whether the absolute value of the correlation exceeded the threshold value of 0.35. We used a threshold value of 0.35 because it represented a point of inflection in the number of nonzero entries in the transformed correlation matrix (Figure S1). The transformed correlation matrix had a total of 184 nonzero entries (130 positive and 54 negative), and only seven of the 54 species did not have a nonzero entry with any other species in the community.

### 2.4 Modeling environmental co‐responses among species as independent variables (sGLM)

The sGLM included the occurrence of nonfocal species as additional independent variables:

(2) $$Y_{\mathrm{ij}}∼T_{j}+P_{j}+V_{j}^{T}+L_{j}+\sum_{i^{\prime }\neq i}Y_{i^{\prime }j}$$

where the final summation term only includes species that have been shown to strongly influence the occurrence of species *i* according to the correlation matrix (note that each nonfocal species *i*′ has a unique GLM slope coefficient)—this ensures that the sGLM describes the same environmental co‐responses as the eGLM + BN, described below. For species without any modeled co‐response terms, the eGLM, sGLM, and eGLM + BN all give identical results.

### 2.5 Modeling environmental co‐responses among species using a Bayesian network (eGLM + BN)

A BN represents environmental co‐responses as conditional dependencies between the occurrence probabilities of individual species in a community (Staniczenko et al., 2017). Compared to some multi‐species models that include the occurrence of nonfocal species as additional independent variables (e.g., sGLM), the BN is applied as a separate, second step after environment‐only models. We based the BN for the peat bog community on the above correlation matrix of environmental co‐responses among species. In this application, occurrence probabilities from the eGLM, so‐called “prior” probabilities, are combined with the BN to obtain “posterior” probabilities that reflect environmental co‐responses among species.

**Figure 1:**
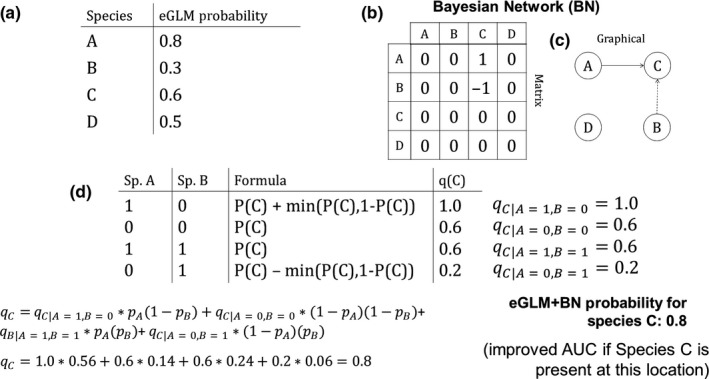
Example workflow for calculating occurrence probabilities using a Bayesian network that represents environmental co‐responses among a community of four species. (a) “Prior” occurrence probabilities for four species at a hypothetical location are first obtained from an eGLM, which takes into account only environmental conditions at a specific location. (b) Correlations between the occurrence of each pair of species at all sampled locations (hypothetical; not shown here) are used to identify strong positive (e.g., A and C) and negative (e.g., B and C) environmental co‐responses among species. (c) A hierarchy of species (A above B, B above C, C above D) is used to determine the direction of each influence, described by the graphical component of the Bayesian network. (d) The second component of the Bayesian network is a conditional probability table for C that specifies how the occurrence of A and B at a location affects the occurrence probability of C, and, below, the calculation of the “posterior” occurrence probability for C at the example location, which now takes into account environmental co‐responses as well as abiotic conditions. Notice that the probability for C with the eGLM + BN is higher than with the eGLM because the probability of A (positive co‐response with C) being present at the location is higher than the probability of B (negative co‐response with C). For species with 1 incoming BN edge, there are only 2 distinct conditional probabilities

The BN must be a directed acyclic graph, meaning that (a) directed edges representing conditional dependencies point from one species to another and (b) there is no way of returning to a species by following a sequence of directed edges originating from that species (Staniczenko et al., 2017). To satisfy these criteria, we implemented a hierarchy for the 54 species such that directed edges point from species higher up in the hierarchy to those lower down. We used a hierarchy based on species abundance (aggregated across the 56 locations), with directed edges pointing from more abundant to less abundant species. Starting with the transformed correlation matrix, we removed any nonzero entries associated with edges that pointed from a less abundant to more abundant species. The result was a BN with 65 positive and 27 negative conditional dependencies involving 47 of the 54 species (Figure S2). We used the Boolean “OR” rule to determine how prior probabilities from the eGLM are converted to posterior probabilities when a species has multiple conditional dependencies in the BN (Staniczenko et al., 2017) (see Figure 1 for a worked example).

### 2.6 Evaluating model performance

We evaluated the effect of data availability on model performance by using a fraction of the empirical data in a training partition to calibrate models and the remaining data in a test partition to measure predictive accuracy. We considered three proportions of training and test partition sizes: 25% (14 of 56) training and 75% test, 50% (28 of 56) training and 50% test, and 75% (42 of 56) training and 25% test. We ran 1,000 randomizations of data for each proportion. We measured the predictive accuracy of each model using the area under receiver operating characteristic curve (AUC) method, which measures the ability of an SDM to discriminate between known species presences and absences (Jiménez‐Valverde, 2012). We also considered the true skill statistic (Allouche, Tsoar, & Kadmon, 2006) (TSS). We considered AUC and TSS due to their relative popularity, but other measures of model performance are available (Fernandes, Scherrer, & Guisan, 2019; Lobo, Jimenez‐Valverde, & Real, 2007; Peterson, Papes, & Soberon, 2014). We found that TSS resulted in such high variability between randomizations (Figure S3) that we could not distinguish the performance of the different models as easily as with AUC.

To obtain an upper bound to model performance, we modified a joint species distribution model (Ovaskainen et al., 2010) (JSDM) that attempts to quantify a potential relationship between every pair of species in a community. Our JSDM‐inspired approach represents the probability of occurrence of a species as a random variable in a jointly distributed set of normal random variables; that is, co‐occurrence relationships between species are described by correlations between random variables. Each component of this multivariate distribution—one univariate normal random variable representing one species—is centered at the original eGLM estimate for a species; that is, with no correlations between random variables this approach reduces to a set of independent eGLMs. At the multivariate level, these correlations are organized into a symmetric correlation matrix containing values between −1 and 1. We used a 54‐by‐54 species correlation matrix to quantify the strength of potential co‐responses between species. Very few species are totally uncorrelated, so the distribution of each component depends on the value of the other components. From a statistical standpoint, this means that we can draw from conditional probability theory to obtain a revised distribution for each species given the known values of the others (Bischoff & Feiger, 1991). In other words, the probability that a species is present at a particular location requires knowing the occurrences of all other species at that location. While the original JSDM (Ovaskainen et al., 2010) used the correlation matrix to predict an entire set of components at once (essentially simulating for all species at once from a single random multivariate distribution), we generated occurrence predictions for one species at a time by combining its original estimate from the eGLM with its correlations with all other species in the community.

The amount of information contained in the JSDM‐inspired approach means it is expected to produce very good predictions. But the large amounts of data required for parametrization compared to the eGLM, sGLM, and eGLM + BN means its output should be considered a practically unattainable upper bound. While the JSDM requires data on every species in the community, the sGLM and eGLM + BN only require data on species for which notable environmental co‐responses are thought to exist. Of course, the eGLM does not require data on any other species to make predictions on the occurrence of a target species. In short, the JSDM‐inspired model treats a community as a being fully connected, while the eGLM + BN and the sGLM attempt to identify the most parsimonious set of interspecific relationships, saving on the expense of data collection and computational time. The data requirements of each model are summarized in Table S3.

### 2.7 Identifying co‐responsive species whose occurrence patterns are strongly influenced by other species

We identified a group of species whose occurrence predictions were greatly improved by the addition of the BN. We measured the overall benefit the BN added to environment‐only models using ΔAUC, which we defined as the difference in AUC scores between the eGLM and the eGLM + BN for an individual species when data were separated into 50% training and 50% test partition sizes. We ran 10 sets of 100 randomizations, considering species with ΔAUC above 0.08 in at least nine of the 10 sets to be “co‐responsive species” (Table S4). We used boosted regression tree analysis (Elith, Leathwick, & Hastie, 2008; de Ville, 2013) to investigate the shared properties of co‐responsive species. Boosted regression tree analysis assigned a “relative importance” to six species‐specific properties according to each property's ability to explain ΔAUC values among co‐responsive species (see Appendix S1); relative importance values across all properties sum to one. We boosted 1,000 trees to measure the relative importance of the six properties, using a sample size of 54 (the number of species in the community) as the input data. The six species‐specific properties we considered were as follows: the number of incoming BN edges, the proportion of locations where species occurred (“rarity”), the average abundance at locations where each species occurred, the average eGLM AUC score, whether a species was a vascular plant or a moss belonging to the *Sphagnum* genus, and topological importance. Topological importance is a summary statistic used in graph theory to evaluate the contribution of each node (in this case, each species) to the overall connectedness of the graph; it has been used to determine keystone species in ecological communities (Jordán, Liu, & Davis, 2006).

### 2.8 Identifying predictor species that improve occurrence predictions of other species

We identified a group of “predictor species” that had a strong effect on the occurrence probabilities of co‐responsive species. We defined predictor species as having at least one of the two properties: (a) outgoing BN edges directly connected to two or more co‐responsive species or (b) an outgoing BN edge directly connected to a predictor species as defined by (a), that is, these predictor species are one step removed from influencing two or more co‐responsive species.

We compared this set of predictor species to umbrella (Fleishman et al., 2001) and indicator (Azeria et al., 2009) species to gauge the extent of overlap between the three groups in the peat bog community. Umbrella species are defined as those that coexist with a large number of other species, suggesting that they may be able to act as conservation surrogates to rare species (Lambert, 2011; Roberge & Angelstam, 2003). Here, we defined umbrella species as species that occurred at 42 (75%) or more of the 56 locations. This cutoff produced a group of only five species that can be considered as being exceptionally adaptable and widespread. Indicator species are defined as those that only occur in the presence of lots of other species (Podani & Csanyi, 2010). Here, we defined indicator species as species that, on average, co‐occurred at locations with at least 20 other species from the peat bog community. We chose 20 species as a cutoff because only 15 locations (26.8%) were inhabited by this many species.

We measured the collective ability of predictor species to improve model performance by computing AUC scores for the eGLM + BN with a partial BN containing only edges among co‐responsive and predictor species. As with the original BN, we ran 1,000 samples with training partition sizes of 25%, 50%, and 75%. We then compared ΔAUC values between partial and full BNs for each co‐responsive species.

## 3 RESULTS

### 3.1 Predicting species occurrence at specific locations

**Figure 2:**
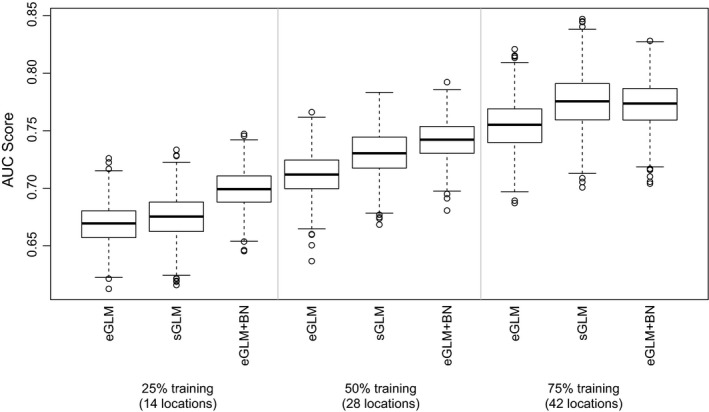
Performance of the eGLM, sGLM, and eGLM + BN measured by AUC at three training partition sizes. The sGLM and eGLM + BN both outperform the eGLM at all partition sizes (1,000 random partitions of the 56 locations for each combination of training partition size and model used). With 25% training data, the eGLM + BN yielded an average AUC score of 0.698, compared to the sGLM average of 0.675 and the eGLM average of 0.668. With 50% training data, the eGLM + BN yielded an average AUC score of 0.741, compared to the sGLM average of 0.730 and the eGLM average of 0.711. With 75% training data, the eGLM + BN yielded an average AUC score of 0.772, compared to the sGLM average of 0.775 and the eGLM average of 0.754. As expected, AUC scores for all models increased in line with the amount of data used for model training. With an unrealistic amount of data available for prediction, we observed AUC scores of 0.848 ± 0.042, 0.844 ± 0.032, and 0.817 ± 0.030 at 75%, 50%, and 25% training, respectively (mean ± *SD*)

We found that modeling environmental co‐responses using the two multi‐species models consistently improved predictions of species occurrence relative to the eGLM. The eGLM + BN performed better than the sGLM when fewer data were used for model training, but the sGLM performed better when more data were used for model training (Figure 2). When using TSS to evaluate model performance, trends were similar but the difference between the models was less pronounced (Figure S3). Because of this result, we used AUC as our primary measure of model performance.

The eGLM + BN improved predictions (ΔAUC > 0) for almost every species in the peat bog community (Table S6). We focused further analysis on this model to better understand its increased prediction accuracy with limited amounts of calibration data compared to the eGLM and sGLM (Figure 2). Aside from the 14 species without any incoming BN edges (by definition the BN does not modify predictions for these species), ΔAUC values were positive for all but six species; the remaining 40 species had an average ΔAUC value of 0.040 ± 0.041 (mean ± *SD*), and only five of these species had ΔAUC values below 0.01.

### 3.2 Characterizing co‐responsive species

Of the 54 species from the peat bog community, we identified six species with ΔAUC values consistently above 0.08, indicating that the eGLM + BN was particularly effective at improving predictions for these species. We used boosted regression tree analysis (Elith et al., 2008) to investigate the shared properties of these co‐responsive species. We found that rarity had the highest relative importance value of the six species‐specific properties we considered (Table 1). This result suggests that co‐responsive species are characterized as being rare—indeed, they occurred at an average of only 11.6% (six of 56) of the locations, compared to the community‐wide average of 34.1% (19 of 56). We explored whether this finding may have arisen due to our use of an abundance‐based hierarchy to specify the direction of BN edges, but further analysis showed that this choice of hierarchy was not responsible for the result that co‐responsive species are typically rare species (see Appendix S1). Five of the six co‐responsive species were particularly rare (occurring at less than 15% of the locations). The next most important property was the eGLM AUC average for the species, suggesting that the BN is especially beneficial when environmental variables on their own provide relatively poor predictions of species' occurrences. The six co‐responsive species had an average eGLM AUC of 0.665 ± 0.068, compared to the overall average of 0.710 ± 0.105.

**Table 1:** Relative importance of six properties associated with species according to boosted regression tree analysis

Property	Relative importance (%)
Rarity	34.9
Average eGLM AUC score with 50% training data	24.2
Average abundance	18.5
Number of incoming BN edges	15.8
Topological importance	6.1
*Sphagnum* moss or vascular plant	0.5

Note

Relative importance values sum to 1 and are based on the proportion of decision trees (our boosted regression tree model involves the boosting of 1,000 decision trees to model how ΔAUC varies in response to changes in the six predictor variables below) that include each predictor variable.

### 3.3 Characterizing predictor species

We identified eight predictor species that had a strong effect on the occurrence probabilities of co‐responsive species. Two of the predictor species had multiple outgoing BN edges pointing directly to co‐responsive species, while the other six indirectly influenced co‐responsive species through BN edges with the first type of predictor species. (One of the co‐responsive species, *Vaccinium vitis‐idea*, actually met the criteria for a predictor species by having two outgoing BN edges pointing toward other co‐responsive species, but we chose to consider it only as a co‐responsive species in subsequent analysis). Predictor species generally had high eGLM AUC scores and low ΔAUC values. The average eGLM AUC score for predictor species was 0.754 ± 0.123 with 50% training data, higher than the overall average of 0.710 ± 0.105. Predictor species had an average ΔAUC value of 0.009 ± 0.016, lower than the overall average of 0.029 ± 0.040 and much lower than the co‐responsive species, which had an average of 0.114 ± 0.020. Taken together, these results suggest that predictor species are relatively insensitive to the presence or absence of other species and their occurrences are well predicted by abiotic conditions alone. Predictor species were more common than usual but not especially widespread; on average, each predictor species occurred at 45.1% (25 of 56) locations.

**Figure 3:**
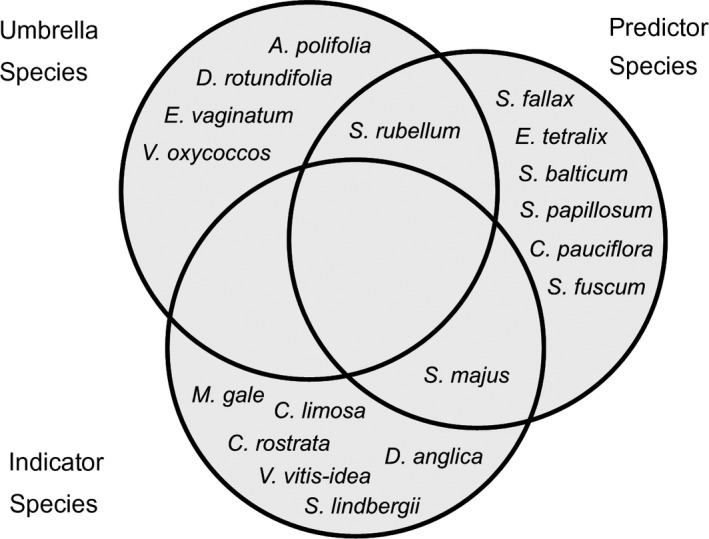
Venn diagram summarizing the overlap between umbrella, indicator, and predictor species. Notice that each group has a similar number of species but very few species belong to more than one group

Predictor species appear to be a distinct group from umbrella and indicator species (Figure 3), making them a useful new category of conservation surrogate. In addition to the eight predictor species we identified, we found five umbrella species and seven indicator species in the peat bog community. Only one species from each group was also classified as a predictor species in our community, indicating that they are almost entirely distinct categories of conservation surrogate.

### 3.4 Analyzing a partial Bayesian network of co‐responsive and predictor species

**Figure 4:**
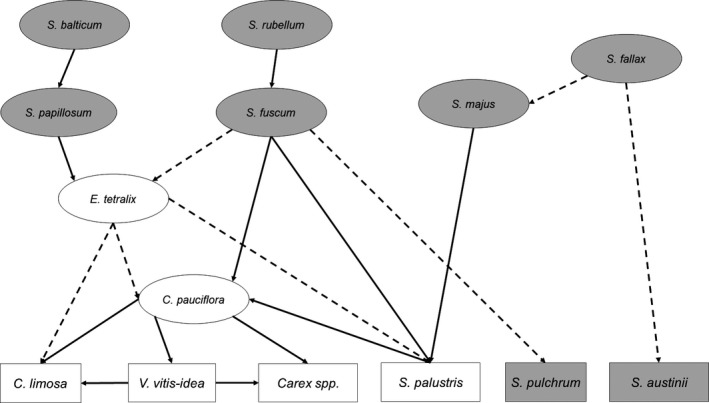
Graphical component of the partial Bayesian network that only includes strong interspecific relationships between predictor species (ovals) and co‐responsive species (rectangles). Solid lines represent positive co‐responses, and dashed lines represent negative co‐responses. *Sphagnum* mosses are shaded gray

We investigated the performance of a BN containing only edges among co‐responsive and predictor species (Figure 4). The partial BN was highly connected with multiple pathways of influence between species. For example, *Sphagnum fallax* (a predictor species) had only one edge pointing directly to a co‐responsive species, yet it indirectly influenced five of the six co‐responsive species. The partial BN generally yielded better AUC scores than the original BN at all three training partition sizes (Table S5), despite the partial BN retaining only 19 (12 positive and seven negative) of the 92 edges in the original BN (including only nine of 17 edges pointing directly to co‐responsive species). Compared to the original BN, which produced ΔAUC values of 0.117 ± 0.065 (mean ± *SD*) for the co‐responsive species, the partial BN produced ΔAUC values of 0.147 ± 0.068 (Table S5). Compared to the original BN, the reduced nature of the partial BN made species occurrence probabilities much easier to compute, while also lowering variability and noise caused by unnecessary BN edges.

## 4 DISCUSSION

We have shown that modeling environmental co‐responses among species from a European peat bog community improved the predictions of rare species occurrence. Based on a BN for the community, we identified two groups of species: co‐responsive species that are typically rare and whose occurrence depends on the presence or absence of other species in the community, and predictor species that are more common and can be used to improve predictions of rare species occurrence. We analyzed a partial BN of only co‐responsive and predictor species and found that this highly connected subnetwork accounts for almost all of the performance of the original BN. This finding suggests that only a small fraction of species and interspecific relationships, particularly those involving predictor species, need to be sampled to improve predictions for multiple rare species in an ecological community.

### 4.1 Comparison of models

Notably, AUC scores for the eGLM + BN with 25% training data were similar to AUC scores for the eGLM with 50% training data (this trend was also apparent when comparing the eGLM + BN with 50% training data to the eGLM with 75% training data). This result suggests that using a BN to predict species occurrences can dramatically reduce the amount of data collection required to calibrate models (although verifying the generality of this result would require testing our approach with a larger data set with more locations). If information on environmental co‐responses among species is available or can be estimated, then the eGLM + BN represents a viable method for improving the accuracy of species occurrence and community composition predictions, while adding minimal effort to the standard approach of environment‐only models.

The sGLM can also be used to reduce data collection, but it lacks some advantages of the eGLM + BN. The difference between the sGLM and eGLM + BN is most prominent with rare species, whose environment‐only model parameters may be especially unreliable due to the difficulty in finding locations at which they are known to be present. The sGLM is likely more sensitive to this unreliability because the effects of other species on the focal species are modeled as additional variables in a GLM, meaning that the benefits afforded by this extra information may remain overwhelmed by the baseline poor performance resulting from the environmental variables. By contrast, the eGLM + BN separates the modeling into an environment component (the eGLM part) and an interspecific component (the BN part)—for rare species and limited data, the information in the BN component can dominate the unreliable environment component, leading to comparatively better predictions.

Although the improved predictions produced by the eGLM + BN and sGLM both result from modeling interspecific relationships, each model may be better suited to describing different types of interspecific relationship. Some pairs of species may simply occur in a similar set of locations due to shared habitat preferences (or in a mutually exclusive manner due to different habitat preferences) in ways that are not described by the particular environmental variables included in the eGLM. In other words, we could attribute some predictive improvement resulting from multi‐species models to more selective, hard‐to‐identify habitat preferences that are shared between species. The sGLM, which models the presences of other species in a similar way to environmental variables, should perform better when the set of nonfocal species used in the model are known to have shared habitat preferences. Conversely, some co‐occurrence relationships may be a result of biotic interactions, such as mutualism, competition, or commensalism. Because the effects of biotic interactions are less tied to environmental variables than shared habitat preferences, the eGLM + BN should perform better in these cases.

The exceptional performance of the JSDM‐inspired approach was unsurprising given the amount of information that can be incorporated in this model. However, to achieve this level of performance, a lot of empirical data is required to parameterize a complete and fully quantified correlation matrix. By contrast, the sGLM only requires knowledge of which species affect the presence of a focal species, while the eGLM + BN only requires knowledge on the presence of important interspecific relationships and the sign—positive or negative—of their effects (see Table S3 for a summary of data requirements for each model). Although using a Bayesian network with our simple assumptions about conditional dependencies can sometimes lead to unrealistic conditional probabilities (i.e., a probability of occurrence of 1 or 0 given the presence or absence of another species), such assumptions are unavoidable in a model that seeks to use as little data as possible. In addition to the potential for the eGLM + BN to incorporate greater biological realism (which would hopefully reduce the frequency of these extreme predictions), discussed below, we argue that some lack of realism is permissible from a practical standpoint because it results in improved predictions compared to the eGLM. In many ways, it is remarkable that the eGLM + BN and sGLM get as close as they do to the performance of the JSDM‐inspired approach. Overall, we consider the models in this study as offering a range of options to inform conservation decision‐making.

### 4.2 Interpreting environmental co‐responses among species

*Sphagnum* mosses are essential to the makeup of peat bog habitats because of the role species in this genus have as ecosystem engineers (van Breemen, 1995). These mosses alter the composition of the soil in which they grow to reduce competition with other plants and increase their intake of nutrients and sunlight. This ability to modify the soil content of peat bogs makes *Sphagnum* mosses prime candidates for predictor species. Indeed, even though *Sphagnum* mosses made up only 37.0% of species from the peat bog community, six of the eight predictor species we identified were *Sphagnum* mosses, including *Sphagnum fuscum*, which is a dominant competitor of vascular plants (Svensson, 1995).

Although boosted regression tree analysis did not identify a strong relationship between *Sphagnum* classification and ΔAUC, *Sphagnum* mosses had an average ΔAUC of 0.048 ± 0.040 compared to the non‐*Sphagnum* average of 0.035 ± 0.039, and two of the six co‐responsive species we identified were *Sphagnum* mosses. These less common *Sphagnum* mosses often have very selective microhabitat preferences (Johnson et al., 2014), and to satisfy these preferences, they modify their habitats. But because many other plants cannot grow in the anoxic, low‐nutrient soil favored by *Sphagnum* mosses, the presence of certain vascular plant species can be used as a signal for the absence of rare *Sphagnum* mosses.

Different *Sphagnum* species can also be very competitive, especially given the close proximity in which they live, suggesting that one *Sphagnum* species would be likely to have an effect on the presence or absence of another (Robroek, Limpens, Breeuwer, Crushell, & Schouten, 2007). Because *Sphagnum* mosses dominate and can even change the conditions of their ecosystem, the composition of *Sphagnum* species in peat bog communities can have a significant effect on the bog habitats where they live. In addition, different *Sphagnum* species prefer to occupy different hydrological gradients in bog habitats, so finding pairs of species that commonly occur together could suggest an environmental co‐response related to this hydrological gradient (Robroek et al., 2007).

### 4.3 Limitations of the data set for testing our approach

The peat bog data set used in this study comprises data for 54 plant species at 56 peat bog locations, which is below the typical size used to train and test multi‐species distribution models. Conventional data sets usually involve significantly more sites than species, around ten times as many locations as there are species (Wisz et al., 2008), and lacking such amounts of data can sometimes result in low model performance, especially for models based on logistic regression (Stockwell & Peterson, 2002). In addition, AUC and TSS are both sensitive to random partitioning with relatively few locations (Lobo et al., 2007; Somodi, Lepesi, & Botta‐Dukát, 2017), something we observed with TSS, in particular. Nevertheless, at the outset we outlined the three properties that made the peat bog data set especially valuable for our analysis and we intend to use our initial results to modify our approach for larger, albeit less well‐resolved data sets.

We simplified the available species abundance data to a measure of binary presence or absence to confirm that the eGLM + BN was effective with this more widely available format of species occurrence data. As with maximum entropy‐based models (Filz, Schmitt, & Engler, 2013), adapting the eGLM + BN to work with abundance data, while not as straightforward (Hongmei, Zheng, & Zhiwei, 2005), could provide more insight into its relative performance, as well as improving its versatility and predictive power.

### 4.4 Adapting our approach to other ecological communities and for conservation

For other ecological communities, improving occurrence predictions using our approach would start by selecting a target species or set of species of interest. The next step is to determine which interspecific relationships involving the target species are worth modeling. We suggest two possible options: modeling environmental co‐responses and modeling biotic interactions. As we did here, positive and negative relationships among species could be measured or estimated to identify a set of candidate species whose occurrences are strongly correlated with the target species. Alternatively, a set of candidate species could be developed based on which species have biotic interactions (e.g., competitive, facilitative) with the target species (Staniczenko et al., 2017). The set of candidate species from either option could then be refined by prioritizing species that fit the criteria for predictor species (i.e., species that are relatively common and insensitive to the presence of other species in the community) for inclusion in a BN. Environment‐only models for these predictor and target species could then be combined with the streamlined BN to generate accurate predictions for the target species.

Umbrella species are characterized by their occurrence in a wide range of habitats (Azeria et al., 2009) and are used as conservation surrogates because their distributions often overlap with other species of interest (Ozaki et al., 2006). However, umbrella species are often so widespread that relying on them to identify occurrences of rare species would lead to many false positives (Das & Dey, 2006). Indicator species are characterized by their occurrence in areas with high species richness (Andelman & Fagan, 2000) and are used as conservation surrogates because their presence highlights locations with suitable conditions for a wide variety of species (Siddig, Ellison, Ochs, Villar‐Leeman, & Lau, 2016). However, their presence is not guaranteed to inform the presence of rare species, which may have very different habitat preferences from more common species in the community (Spitale, 2012). Umbrella and indicator species offer a broad overview of ecosystem health and functioning to conservation practitioners (Halme, Mönkkönen, Kotiaho, Ylisirniö, & Markkanen, 2009; Thorne, Cameron, & Quinn, 2006). As with all conservation surrogates, some initial analysis is necessary to identify these groups in a new ecological community (Araújo et al., 2014), but once identified, each group offers distinct benefits for particular aims. Predictor species, which are defined by their relationship to rare species, offer a more detailed and finely resolved perspective that can complement umbrella and indicator species as part of a comprehensive conservation strategy. We hope that in the near future conservationists could use a model like the eGLM + BN to predict more accurately the geographical distributions of rare species and therefore protect more effectively Earth's declining biodiversity.

## CONFLICT OF INTEREST

All authors declare no competing interests.

## AUTHOR CONTRIBUTIONS

W.F. and P.P.A.S. designed the study; P.R.T. wrote code and performed analysis; P.R.T. wrote the first draft and all authors edited the manuscript; all authors have approved the final version of the manuscript.

## Data Availability

Environmental data and species abundance data for the peat bog community at all 56 locations are publicly available as part of a previously published study (https://datadryad.org/stash/dataset/doi:10.5061/dryad.g1pk3)44. A copy of our R code, along with example data, is publicly available on GitHub (https://github.com/pthompson234/predictorspecies).
