# Supplementary figures and images for: Predictor species: Improving assessments of rare species occurrence by modeling environmental co‐responses
Source: Ecol Evol. 2020 Mar 2;10(7):3293–304. doi: 10.1002/ece3.6096 (PMC7140998; doi:10.1002/ece3.6096)

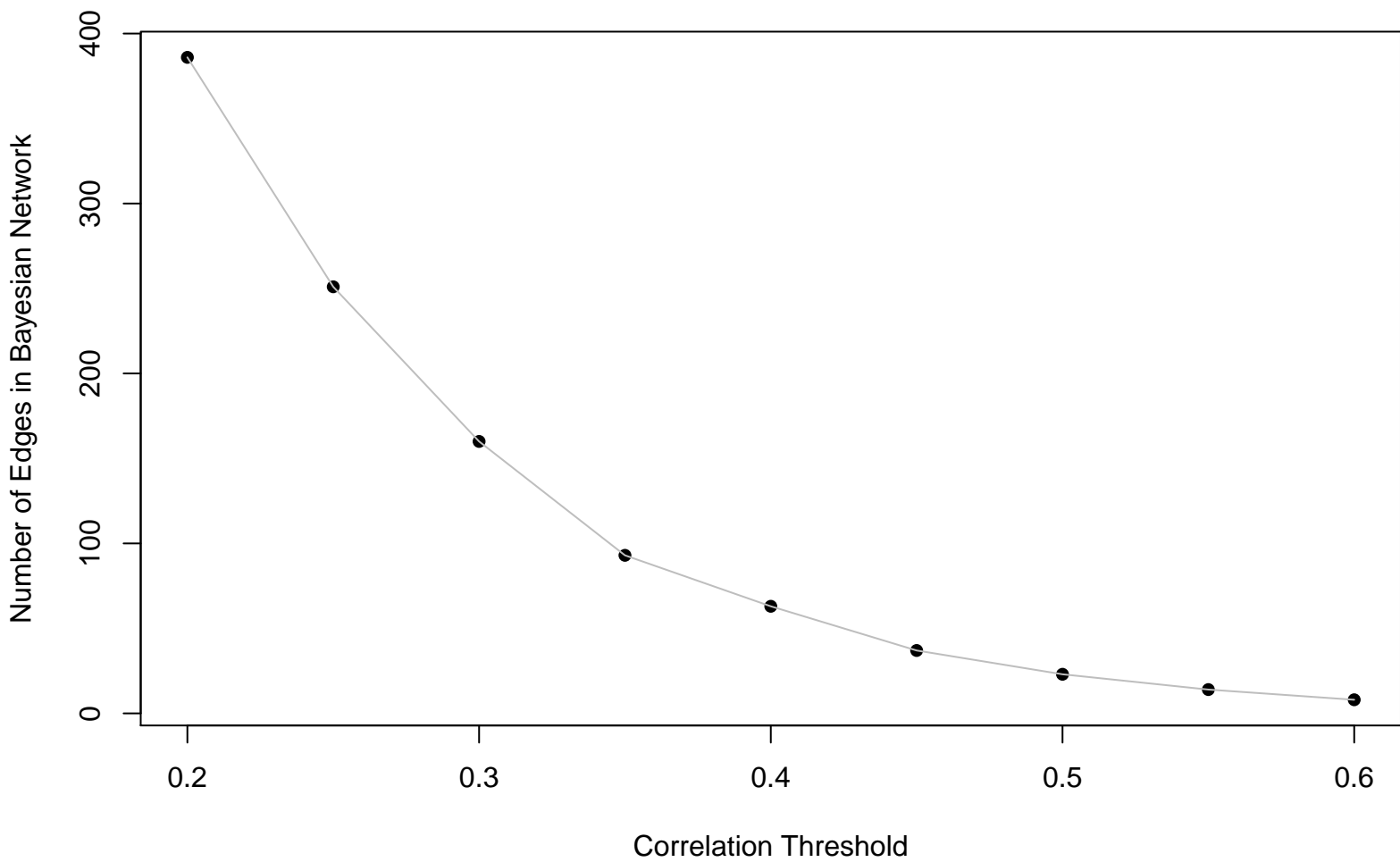

Supplement: Supplementary file 1 [file ECE3-10-3293-s001.pdf]

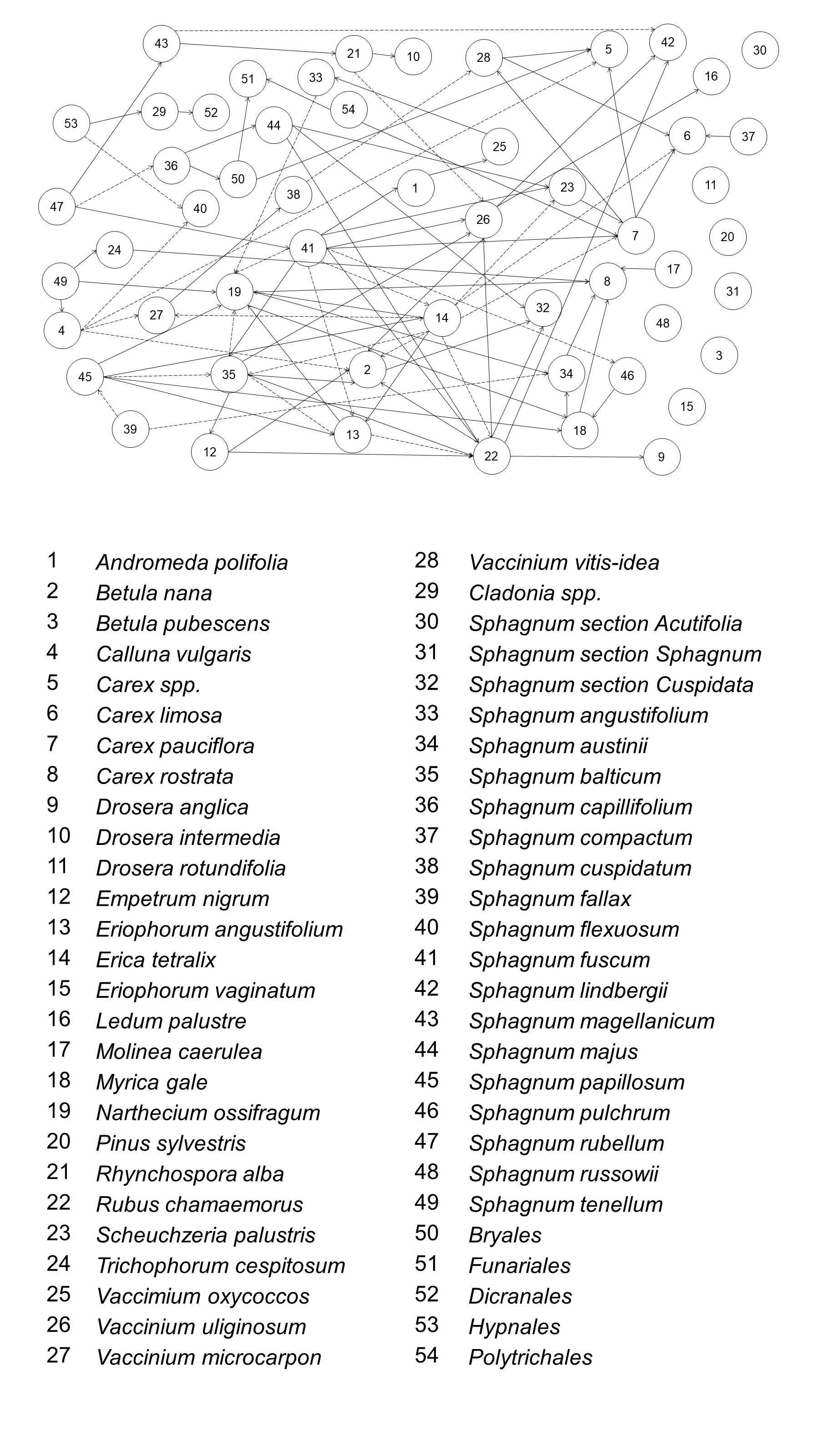

Supplement: Supplementary file 2 [file ECE3-10-3293-s002.tif]

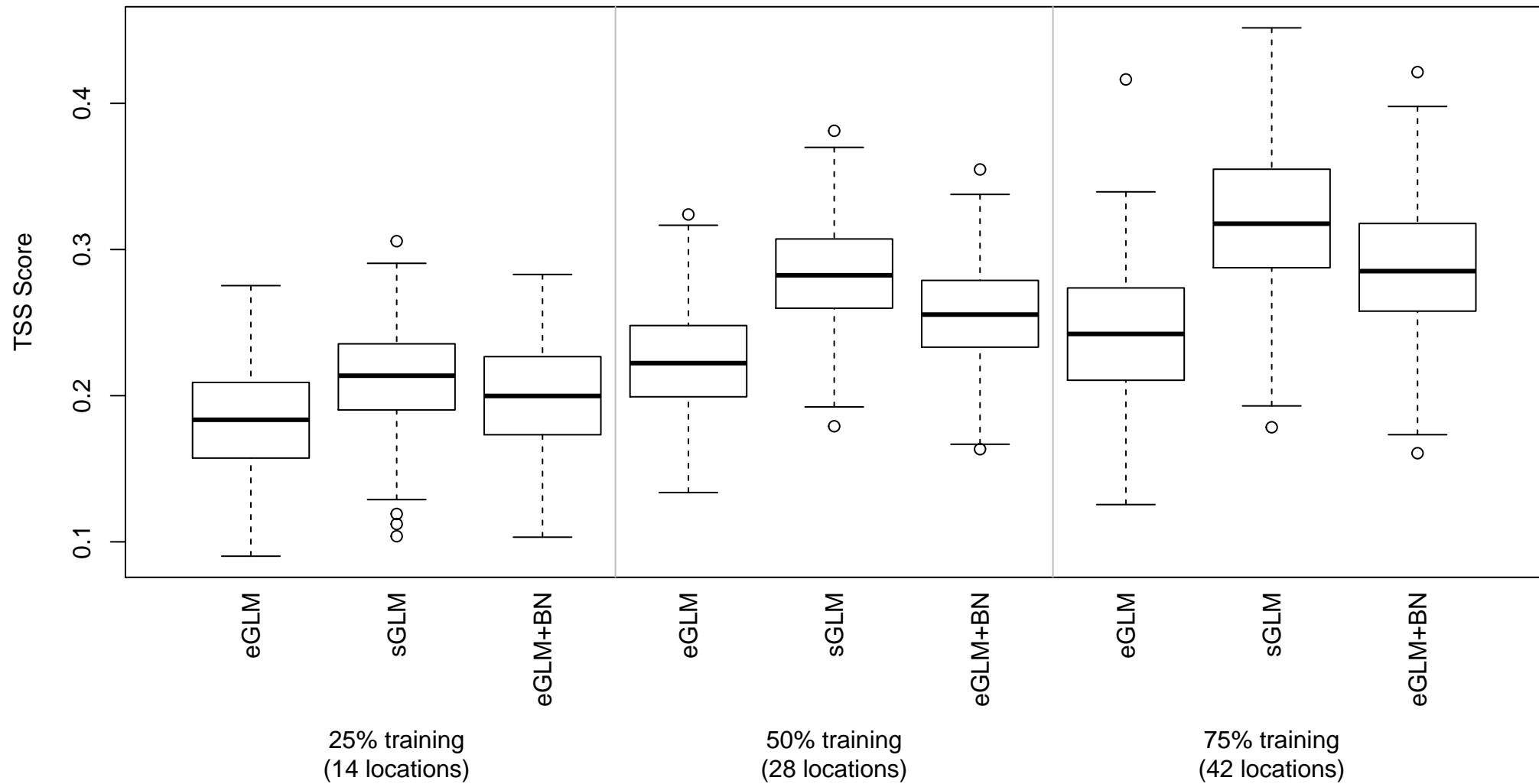

Supplement: Supplementary file 3 [file ECE3-10-3293-s003.pdf]
