# Supplementary material for: Predictor species: Improving assessments of rare species occurrence by modeling environmental co‐responses
Source: Ecol Evol. 2020 Mar 2;10(7):3293–304. doi: 10.1002/ece3.6096 (PMC7140998; doi:10.1002/ece3.6096)
Supplement: Supplementary file 4 [file ECE3-10-3293-s004.docx]

**SUPPLEMENTARY TABLE 1**

| **Environmental Variable** | **Average Correlation with Species Presence/Absence** |
| --- | --- |
| Temperature Seasonality | 0.260 |
| Latitude | 0.253 |
| Mean Annual Temperature | 0.243 |
| Mean Annual Precipitation | 0.200 |
| Longitude | 0.191 |
| Lang’s moisture index | 0.173 |
| Precipitation Seasonality | 0.169 |
| Altitude | 0.169 |
| Reduced nitrogen concentration | 0.164 |
| Atmospheric sulfur concentration | 0.160 |
| Oxidized nitrogen concentration | 0.152 |

**Supplementary Table 1** – **Absolute values of Pearson correlation between each environmental variable and the occurrence of species across all locations, on average (n = 56).** Underlined variables were used in generalized linear models (GLMs) because of their relatively high correlations.
