# Supplementary material for: Predictor species: Improving assessments of rare species occurrence by modeling environmental co‐responses
Source: Ecol Evol. 2020 Mar 2;10(7):3293–304. doi: 10.1002/ece3.6096 (PMC7140998; doi:10.1002/ece3.6096)
Supplement: Supplementary file 5 [file ECE3-10-3293-s005.docx]

**SUPPLEMENTARY TABLE 2**

| **Environmental Variable** | Mean Annual Temperature | Mean Annual Precipitation | Temperature Seasonality | Latitude |
| --- | --- | --- | --- | --- |
| Mean Annual Temperature |  | 0.468 | -0.548 | -0.414 |
| Mean Annual Precipitation |  |  | 0.015 | 0.136 |
| Temperature Seasonality |  |  |  | 0.475 |
| Latitude |  |  |  |  |

**Supplementary Table 2** **– Table depicting correlations between all the environmental variables used in the eGLM and subsequent models**. None of these variables are correlated at an abnormally strong value (the highest being roughly 0.55), suggesting that there are no overfitting issues in the eGLM, sGLM or eGLM+BN. Obviously the variables have some sort of relationship, but since none of the correlations are remotely linear for any pair of environmental variables, each variable is important in the construction of GLMs for species occurrence predictions.
