# Supplementary material for: Predictor species: Improving assessments of rare species occurrence by modeling environmental co‐responses
Source: Ecol Evol. 2020 Mar 2;10(7):3293–304. doi: 10.1002/ece3.6096 (PMC7140998; doi:10.1002/ece3.6096)
Supplement: Supplementary file 6 [file ECE3-10-3293-s006.docx]

**SUPPLEMENTARY TABLE 3**

| **Model** | **Data requirements** |
| --- | --- |
| eGLM | - Environmental data on each location in the community - Presences/absences for all species at locations in the training partition |
| sGLM | - Environmental data on each location in the community - Presences/absences for all species at locations in the training partition - Knowledge of any interspecific interactions with the focal species (co-occurrence relationships, biotic interactions, etc.) - Presences/absences for species interacting with the focal species at all locations |
| eGLM+BN | - Environmental data on each location in the community - Presences/absences for all species at locations in the training partition - Knowledge of any interspecific interactions with the focal species (co-occurrence relationships, biotic interactions, etc.) and some knowledge about the nature of these interactions (positive/negative) - Presences/absences for species interacting with the focal species at all locations |
| JSDM-inspired approach | - Environmental data on each location in the community - Presences/absences for all species at locations in the training partition - Presences/absences for all species at locations in the test partition - Exact correlation values for each pair of species in the community (more than just a knowledge of interactions) |

**Supplementary Table 3** – **Table describing the data requirements for each model.** Obviously the eGLM, which does not incorporate species interactions, requires the smallest amount of data, but for multi-species models, both the sGLM and eGLM+BN do not require very much additional data. The approach inspired by joint species distribution modelling (JSDM) techniques requires data that may be difficult or impossible to collect in some situations, making it a potentially unrealistic model choice. As a result, we focus on how the eGLM+BN or sGLM can be used to facilitate improved conservation practices with a much lower amount of input data.
