# Supplementary material for: Predictor species: Improving assessments of rare species occurrence by modeling environmental co‐responses
Source: Ecol Evol. 2020 Mar 2;10(7):3293–304. doi: 10.1002/ece3.6096 (PMC7140998; doi:10.1002/ece3.6096)
Supplement: Supplementary file 7 [file ECE3-10-3293-s007.docx]

**SUPPLEMENTARY TABLE 4**

| **Number of random sets, η** | **Number of species with ΔAUC > 0.08 in at least η sets** |
| --- | --- |
| 1 | 10 |
| 2 | 9 |
| 3 | 8 |
| 4 | 8 |
| 5 | 8 |
| 6 | 7 |
| 7 | 6 |
| 8 | 6 |
| 9 | 6 |
| 10 | 3 |

**Supplementary Table 4** – **Table describing the prevalence of high ΔAUC (change in AUC score average from eGLM to eGLM±BN at 50% training partition) values in the different random sets, while also explaining the variability in each set.** Species with ΔAUC above 0.08 in at least 9 of the 10 random sets (shaded in light gray) were classified as co-responsive species; we identified 6 co-responsive species in the peat bog plant community.
