# Supplementary material for: Predictor species: Improving assessments of rare species occurrence by modeling environmental co‐responses
Source: Ecol Evol. 2020 Mar 2;10(7):3293–304. doi: 10.1002/ece3.6096 (PMC7140998; doi:10.1002/ece3.6096)
Supplement: Supplementary file 8 [file ECE3-10-3293-s008.docx]

**SUPPLEMENTARY TABLE 5**

| **Species** | **Rarity (%)** | **ΔAUC with partial BN** | **ΔAUC with original BN** |
| --- | --- | --- | --- |
| *Carex spp.* | 8.93 | 0.124 ± 0.232 | 0.134 ± 0.178 |
| *Carex limosa* | 10.71 | 0.147 ± 0.141 | 0.099 ± 0.165 |
| *Vaccinium vitis-idea* | 9.72 | 0.130 ± 0.166 | 0.097 ± 0.153 |
| *Scheuchzeria palustris* | 17.86 | 0.128 ± 0.106 | 0.126 ± 0.137 |
| *Sphagnum austinii* | 8.93 | 0.199 ± 0.093 | 0.088 ± 0.174 |
| *Sphagnum pulchrum* | 12.50 | 0.153 ± 0.135 | 0.139 ± 0.114 |

**Supplementary Table 5** – **ΔAUC values (mean ± standard deviation) for the six co-responsive species with partial and original Bayesian networks (BNs).** Note the similarity, and in many cases, improvement, between ΔAUC values for each species. We also include rarity values to display how co-responsive species generally occur at an exceptionally low proportion of the 56 peat bog sites.
