# Supplementary material for: Predictor species: Improving assessments of rare species occurrence by modeling environmental co‐responses
Source: Ecol Evol. 2020 Mar 2;10(7):3293–304. doi: 10.1002/ece3.6096 (PMC7140998; doi:10.1002/ece3.6096)
Supplement: Supplementary file 9 [file ECE3-10-3293-s009.docx]

**SUPPLEMENTARY TABLE 6**

| **Species Name** | **eGLM AUC** | **sGLM-eGLM AUC** | **ΔAUC** |
| --- | --- | --- | --- |
| *Andromeda polifolia* | 0.785 ± 0.130 | 0.005 ± 0.114 | 0.026 ± 0.111 |
| *Betula nana* | 0.820 ± 0.099 | -0.110 ± 0.117 | 0.039 ± 0.127 |
| *Carex spp* | 0.632 ± 0.203 | 0.178 ± 0.223 | 0.134 ± 0.178 |
| *Carex limosa* | 0.690 ± 0.157 | 0.036 ± 0.196 | 0.099 ± 0.165 |
| *Carex pauciflora* | 0.803 ± 0.122 | 0.002 ± 0.141 | 0.029 ± 0.149 |
| *Carex rostrata* | 0.816 ± 0.216 | -0.017 ± 0.180 | -0.003 ± 0.169 |
| *Drosera anglica* | 0.653 ± 0.095 | 0.051 ± 0.113 | 0.044 ± 0.113 |
| *Drosera intermedia* | 0.642 ± 0.163 | 0.099 ± 0.134 | 0.067 ± 0.124 |
| *Empetrum nigrum* | 0.876 ± 0.077 | -0.004 ± 0.045 | -0.018 ± 0.095 |
| *Eriophorum angustifolium* | 0.826 ± 0.083 | -0.025 ± 0.102 | 0.046 ± 0.120 |
| *Erica tetralix* | 0.868 ± 0.079 | -0.036 ± 0.059 | 0.010 ± 0.109 |
| *Ledum palustre* | 0.861 ± 0.198 | -0.027 ± 0.154 | 0.030 ± 0.140 |
| *Myrica gale* | 0.871 ± 0.157 | 0.038 ± 0.126 | -0.004 ± 0.147 |
| *Narthecium ossifragum* | 0.909 ± 0.085 | -0.019 ± 0.114 | 0.000 ± 0.169 |
| *Rhynchospora alba* | 0.678 ± 0.080 | 0.039 ± 0.054 | 0.063 ± 0.082 |
| *Rubus chamaemorus* | 0.907 ± 0.080 | -0.076 ± 0.102 | 0.001 ± 0.130 |
| *Scheuchzeria palustris* | 0.618 ± 0.105 | 0.176 ± 0.145 | 0.126 ± 0.137 |
| *Trichophorum cespitosum* | 0.690 ± 0.095 | 0.066 ± 0.070 | 0.051 ± 0.099 |
| *Vaccimium oxycoccos* | 0.695 ± 0.110 | 0.021 ± 0.099 | 0.014 ± 0.103 |
| *Vaccinium uliginosum* | 0.803 ± 0.116 | -0.022 ± 0.124 | 0.035 ± 0.143 |
| *Vaccinium microcarpon* | 0.750 ± 0.142 | 0.005 ± 0.113 | -0.037 ± 0.125 |
| *Vaccinium vitis-idea* | 0.674 ± 0.169 | 0.076 ± 0.177 | 0.097 ± 0.153 |
| *Cladonia spp* | 0.675 ± 0.086 | 0.026 ± 0.058 | 0.036 ± 0.073 |
| *Sphagnum section Cuspidata* | 0.638 ± 0.238 | -0.012 ± 0.184 | 0.027 ± 0.163 |
| *Sphagnum angustifolium* | 0.674 ± 0.087 | 0.032 ± 0.070 | 0.038 ± 0.081 |
| *Sphagnum austinii* | 0.797 ± 0.196 | -0.022 ± 0.166 | 0.088 ± 0.174 |
| *Sphagnum balticum* | 0.966 ± 0.046 | -0.014 ± 0.060 | -0.001 ± 0.103 |
| *Sphagnum capillifolium* | 0.582 ± 0.079 | 0.064 ± 0.102 | 0.079 ± 0.096 |
| *Sphagnum compactum* | 0.824 ± 0.278 | -0.051 ± 0.152 | 0.014 ± 0.158 |
| *Sphagnum flexuosum* | 0.620 ± 0.146 | 0.062 ± 0.172 | 0.069 ± 0.137 |
| *Sphagnum fuscum* | 0.808 ± 0.146 | 0.004 ± 0.053 | -0.016 ± 0.077 |
| *Sphagnum lindbergii* | 0.803 ± 0.205 | 0.002 ± 0.171 | 0.038 ± 0.171 |
| *Sphagnum magellanicum* | 0.693 ± 0.099 | 0.058 ± 0.080 | 0.066 ± 0.104 |
| *Sphagnum majus* | 0.642 ± 0.093 | 0.029 ± 0.072 | 0.038 ± 0.091 |
| *Sphagnum papillosum* | 0.719 ± 0.084 | 0.000 ± 0.055 | 0.013 ± 0.076 |
| *Sphagnum pulchrum* | 0.583 ± 0.107 | 0.094 ± 0.124 | 0.139 ± 0.114 |
| *Sphagnum tenellum* | 0.583 ± 0.087 | 0.066 ± 0.100 | 0.086 ± 0.097 |
| *Bryales* | 0.592 ± 0.085 | 0.070 ± 0.148 | 0.005 ± 0.112 |
| *Funariales* | 0.627 ± 0.124 | 0.107 ± 0.170 | 0.008 ± 0.152 |
| *Dicranales* | 0.640 ± 0.080 | 0.035 ± 0.070 | 0.019 ± 0.078 |

**Supplementary Table 6 – eGLM AUC values, ΔAUC (eGLM+BN-eGLM) values and (sGLM-eGLM) AUC values (mean ± standard deviation) for every taxon in the peat bog community that had incoming BN edges (i.e., cases where the multi-species models could be used to potentially improve eGLM predictions) at the 50% training partition.** This data comes from 1,000 random partitions of the 56 locations in the peat bog community. Co-responsive species are shaded gray.
