# Supplementary material for: Predictor species: Improving assessments of rare species occurrence by modeling environmental co‐responses
Source: Ecol Evol. 2020 Mar 2;10(7):3293–304. doi: 10.1002/ece3.6096 (PMC7140998; doi:10.1002/ece3.6096)
Supplement: Supplementary file 10 [file ECE3-10-3293-s010.docx]

**APPENDIX 1: SUPPLEMENTARY METHODS AND FIGURES**

**Modifying data for Bayesian network analysis**

We modified raw plant abundance data from peat bogs in Europe to use for generalized linear modelling, which requires the response variable to be on a binomial scale^1^. We acquired raw data in the form of two files, the first being a file containing abundance values for 54 plant taxa at 56 peat bog locations^2^. The authors of the study from which we obtain our data grouped together rare and closely related species (e.g., rare members of the *Sphagnum cuspidatum* subgenus) into one taxon to lower the number of very sparse (i.e., only present at one location) taxa^2^. We based all our occurrence prediction models on generalized linear models (GLMs), which require the choice of a specific link function; since we chose a logit link, we needed all the species occurrence data to be on a binomial ([0,1]) scale.

We were also given a data table containing environmental data for each of the European peat bog locations^2^, which we used to calibrate our GLMs. Eleven variables were available for use in models: altitude, longitude, latitude, mean annual temperature, mean annual precipitation, temperature seasonality, precipitation seasonality, atmospheric deposition of sulfur, oxidized and reduced nitrogen, and Lang’s moisture index, which is the ratio of precipitation to temperature in the warmest month in the year^2^. We limited our set of independent variables to those that are strongly related to the habitat preferences of plants. We identified which variables should be used in these models by computing Pearson correlation between each variable and species presence or absence (Table S1). We used mean annual temperature, latitude, mean annual precipitation, and temperature seasonality because these variables had relatively strong correlations with the presence or absence of the plant species in the peat bog community.

The environmental variables we chose were not highly correlated with each other, indicating that all of them provide unique value to the regression; none of the values had a correlation with absolute value above 0.55 (Table S2). We suggest that this result is likely because we used data from a study in Europe, an area with a variety of different climates (the Mediterranean region of Europe is quite warm relative to other areas with similar latitudes) and some surrounding islands (the British Isles, like most islands, likely have different climatic conditions than the nearby mainland).

**Generalized linear models**

We used R to generate GLMs for each plant species based on a set of random training partitions of locations^3^. The R “glm” function allows us to create individual GLMs for each species based on a subset of the 56 peat bog locations, and use these GLMs to predict the occurrence of these species at the rest of the locations. We used a data table containing the presence or absence of a species at each location, along with the four environmental variables, as data for GLMs. We used the “predict” function on a set of numeric entries representing the bioclimatic conditions at each location in the test partition. The result was a vector of probabilities for each species at all the locations in the test partition, allowing us to compare probabilities from our GLMs to other models as well as to themselves. In the sGLM, we used the presence (or absence) of potentially influential other species as an additional set of independent variables; the sum of these terms was not correlated with any of the environmental variables (correlations between this sum and the four environmental variables were 0.019 for latitude, -0.061 for mean annual temperature, 0.122 for temperature seasonality, and 0.029 for mean annual precipitation).

We tested the accuracy of environment-only GLMs that included quadratic and interaction terms, finding that these terms did not improve model performance and therefore are not necessary. We compared the accuracy of these models using AUC, finding that GLMs with quadratic and interaction terms underperformed the eGLM at all training partition sizes. With 25% training data, the eGLM yielded an average AUC score of 0.668; the GLM with quadratic terms yielded an average AUC score of 0.645 and the interaction GLM yielded an average AUC score of 0.630. With 50% training data, the original eGLM, the quadratic GLM, and the interaction GLM yielded AUC score averages of 0.711, 0.683, and 0.660, respectively. With 50% training data, the original eGLM, the quadratic GLM, and the interaction GLM yielded AUC score averages of 0.754, 0.734, and 0.719, respectively.

**Using a BN to identify environmental co-responses among plant species**

We created a binary, symmetric correlation matrix to use as a basis for the BN. We evaluated Pearson correlation values for each combination of plant species, and displayed the results in a similar, 54 x 54 matrix. We analyzed the effect that different correlation thresholds would have on the number of edges in the resulting BN to choose the optimal threshold (Figure S1). The finalized correlation matrix exclusively contains entries of 0, 1 and -1. With a large number of entries eliminated entirely, and any significant correlations rendered as equal, this resulting output is much more simple than a true correlation matrix.

Because the correlation matrix is symmetric and a BN cannot be, we invoked a hierarchy that directed the co-responses in the correlation matrix. Our hierarchy was based on the total abundance of each species over all 56 locations; with this hierarchy, individual species can only have incoming BN edges pointing from more abundant species. While we understand that this is likely not the nature of every interspecific interaction in the community, this hierarchy works generally well for a community that we know little about beyond our basic abundance data.

We evaluated eGLM+BN probabilities using the Boolean “OR” rule, which follows the following formula for a species *i* (Figure 1, main text):

$$p_{i, j}^{*}= \left\{ \begin{matrix} p_{i,j}+min(p_{i,j}, 1- p_{i,j}) & \mathrm{if} n_{+, i, j}> n_{-, i, j} \\ p_{i, j} & \mathrm{if} n_{+, i, j}= n_{-, i, j} \\ p_{i, j}-min(p_{i,j}, 1- p_{i,j}) & \mathrm{if} n_{+, i, j}< n_{-, i, j} \end{matrix} \right.$$

where $p_{i, j}^{*}$ is the posterior probability of species *i* at location *j*, and $p_{i, j}$ is the prior probability of species *i* at location *j*; $n_{+, i, j}$ and $n_{-, i, j}$ are the number of species with positive and negative BN edges pointing to species *i* that are present at location *j*, respectively^4^.

Using this rule of thumb, we can develop a conditional probability table given prior probabilities for each species and a BN. Consider a community where Species A, B, and C have priors 0.8, 0.3, and 0.6 respectively (Figure 1, main text). If we are given a BN that includes two edges, one positive edge pointing from A to C and one negative edge pointing from B to C, we can use a conditional probability table to more accurately estimate the true presence rate of C. Because C has two incoming BN edges, we generate a conditional probability table for the 4 (2^2^) possible combinations of presence for Species A and B (A present only, B present only, both present or both absent). For each of these hypothetical scenarios we can calculate $n_{+, C}$ and $n_{-, C}$. When Species A is present and Species B is not, $n_{+, C}$ = 1 and $n_{-, C}$ = 0, so the corresponding entry of the conditional probability table is $p_{C}+min(p_{C}, 1- p_{C})$, where $p_{C}$ is 0.6 as previously stated. This results in the first entry of the conditional probability table (corresponding to situations where A is present and B is absent) being 1. The other three entries are calculated similarly using the above equation for the posterior probability, and then once the conditional probability table is complete, the revised posteriors are calculated using Bayes’ formula.

**Developing a joint species distribution model to quantify co-occurrence relationships**

We used joint multivariate logistic regression to produce estimates for a species’ occurrence at a given location using a full correlation matrix for the plant community^5^. We interpreted the presences or absences of each plant in the peat bog community as one component of a normally distributed random vector (a vector where each component is normally distributed around a corresponding component of a mean vector, and each component may depend on the others based on a correlation matrix). Given a mean vector and a correlation matrix, one can obtain random values and analyze their correlation in order to obtain information about interspecific relationships^5,6^; however, we used this distribution differently because we assumed a known presence or absence for every non-focal species in our analysis. We obtained the conditional distribution of one component of the multivariate vector given the values of the other components (this conditional distribution is normal), using this distribution to estimate the probability that a species was present. We developed the mean vector for this distribution based on the eGLM predictions for each species^5^, so the revised conditional distribution given the other values of the components was shifted according to the presence or absence of the other species.

**Interpreting AUC scores from the three occurrence prediction models**

We used the area under the receiver operating characteristic curve (AUC) method to evaluate the predictive accuracy of each model. AUC scores range from 0 to 1; a score of 1 represents a perfectly correct prediction, while a score of 0.5 represents random guessing^7^. It is then very much expected for all AUC scores in our analysis to be greater than 0.5, and given our findings with our JSDM-inspired approach, we cannot expect AUC scores to be greater than 0.85 for more simple models such as the eGLM, sGLM or eGLM+BN. Therefore, a difference in AUC between models of 0.1 or higher is very significant^7^. Therefore, we choose 0.08 as the ΔAUC value that a species needs to consistently attain for consideration as a co-responsive species (Table S4).

We also experimented with true skill statistic (TSS)^8^ as an alternative to AUC for model evaluation. Unlike AUC, TSS values range from -1 to 1, with 0 representing random guessing. We found that while in some cases TSS and AUC produced similar results, the sGLM outperforms the eGLM+BN at all training sizes when we used TSS to evaluate model performance (Figure S3). TSS values were also much more variable from one randomization to another, making it difficult to confirm the significance of any trends we found. For some random partitions of the 56 locations, the average TSS for each species was close to 0.1 for all three models, while other times this average was close to 0.5. With variability this high it is difficult to draw conclusions or identify various trends as statistically significant.

**Identifying which traits were most pivotal in identifying co-responsive species**

Boosted regression tree (BRT) analysis is a prediction method consisting of the boosting of a large set of decision trees. Decision trees, which predict the value of a dependent variable by checking the value of many independent variables in relation to defined cut-off points^9^, are a basic but effective prediction tactic. However, single decision trees are not very smooth, and the cut-off points can be chosen rather arbitrarily; BRT analysis uses many trees and takes a weighted average of their outputs^10^. Individual trees that are more accurate are given higher weights. When hundreds or thousands of trees are incorporated, the prediction becomes much smoother. We used 1,000 trees in our analysis and trained the model randomly with 75% of the data. Literature on BRT analysis recommends smaller training fractions^10^ but because we only had 54 data points, we could not obtain BRT predictions using a smaller value. We calibrated, tested and evaluated BRT models using the R “gbm” package with little computational expense.

BRT analysis suggests that rarity is very important in determining whether a species is likely to have a high ΔAUC. We explored whether this trend may simply be a consequence of the abundance-based hierarchy of influence we used to construct the BN, which prevented highly abundant species from having incoming BN edges. Although individual species abundance across all locations was positively correlated with rarity (*r* = 0.741, *p* < 0.001), BRT analysis showed that rarity still had a much stronger relationship with ΔAUC than abundance or, more importantly, the number of incoming BN edges. If rare species had high ΔAUC values only because they were on the bottom of the hierarchy, any species with many incoming BN edges (or any incoming BN edges, for that matter) would have a high ΔAUC, but this is not the case.

**APPENDIX REFERENCES**

1. Das, S. & Dey, D.K. On Bayesian analysis of generalized linear models using the Jacobian technique. *Am. Stat.,* **60**, 264-268 (2006).
2. Robroek, B.J.M. et al. Taxonomic and functional turnover are decoupled in European peat bogs. *Nat. Comm.,* **8**, 1161-1169 (2017).
3. Vasconcelos, R.P., Le Pape, O., Costa, M.J. & Cabral, H.N. Predicting estuarine use patterns of juvenile fish with Generalized Linear Models. *Estuar. Coast. Shelf S.,* **120**, 64-74 (2013).
4. Staniczenko, P.P.A., Sivasubramaniam, P., Suttle, K.B. & Pearson, R.G. Linking macroecology and community ecology: refining predictions of species distributions using biotic interaction networks. *Ecol. Lett.,* **20**, 693-707 (2017).
5. Ovaskainen, O., Hottola, J., & Siitonen, J. Modeling species co-occurrence by multivariate logistic regression generates new hypotheses on fungal interactions. *Ecology,* **91**, 2514-2521 (2010).
6. Pollock, L.J. et al. Understanding co-occurrence by modelling species simultaneously with a Joint Species Distribution Model (JSDM). *Methods Ecol. Evol.,* **5**, 397-406 (2014).
7. Jiménez-Valverde, A. Insights into the area under the receiver operating characteristic curve (AUC) as a discrimination measure in species distribution modelling. *Global Ecol. Biogeogr.,* **21**, 498-507 (2012).
8. Allouche, O., Tsoar, A. & Kadmon, R. Assessing the accuracy of species distribution models: prevalence, kappa and the true skill statistic (TSS). *J. Appl. Ecol.,* **43**, 1223-1232 (2006).
9. de Ville, B. Decision trees. *Wiley Periodicals*, **5**, 448-455 (2013).

Elith, J., Leathwick, J.R., & Hastie, T. A working guide to boosted regression trees. *J. Anim. Ecol.,* 77, 802-813 (2008).
