# Supplementary material for: Predictor species: Improving assessments of rare species occurrence by modeling environmental co‐responses
Source: Ecol Evol. 2020 Mar 2;10(7):3293–304. doi: 10.1002/ece3.6096 (PMC7140998; doi:10.1002/ece3.6096)
Supplement: Supplementary file 11 [file ECE3-10-3293-s011.docx]

**Figure S1 – Number of edges in a resulting Bayesian network (BN) based on correlation threshold.** Note that a correlation threshold of 0.35 represents a clear point of inflection; values above 0.35 result in a BN that would leave many species without any edges, while values below 0.35 result in a BN that would potentially contain too many frivolous edges.

**Figure S2** – **The full Bayesian network (BN) produced for our peat bog community.** All but seven species (located on the right side of the graph) are included. Continuous lines represent positive edges, while dashed lines represent negative edges. The entire BN has 92 edges in total; 65 are positive and 27 are negative. We also include a legend displaying the names of each species corresponding to numbers on the graph.

**Figure S3 - Performance of the eGLM, sGLM, and eGLM+BN measured by TSS at three training partition sizes.** While some trends regarding the three models are similar regardless of what evaluation tool is used, the sGLM performs better than the eGLM+BN at all training data sizes. TSS scores also vary much more than AUC scores from one randomization to another.
